# Histopathological and Immunohistochemical Findings in Postmortem Lungs from Mexican Patients with Severe COVID-19

**DOI:** 10.3390/ijms27021049

**Published:** 2026-01-21

**Authors:** Laura Guadalupe Chávez Gómez, Diana Gabriela Ríos Valencia, Tania Lucía Madrigal-Valencia, Lilian Hernández Mendoza, Armando Pérez-Torres, Rocio Tirado Mendoza

**Affiliations:** 1 Laboratorio de Patógenos Virales y Células Troncales, Departamento de Microbiología y Parasitología, Facultad de Medicina, National Autonomous University of Mexico, Mexico City CP 04510, Mexico; lauraqfb04@gmail.com (L.G.C.G.); lilianhernandezm83@facmed.unam.mx (L.H.M.); 2 Laboratorio de Inmunobioquímica de Taenia solium, Departamento de Microbiología y Parasitología, Facultad de Medicina, National Autonomous University of Mexico, Mexico City CP 04510, Mexico; riosdg@facmed.unam.mx; 3 Laboratorio de Infectología, Instituto Nacional de Ciencias Médicas y Nutrición Salvador Zubirán, Mexico City CP 14080, Mexico; a.nia.90@hotmail.com; 4 Laboratorio de Filogenia del Sistema Inmune de Piel y Mucosas, Departamento de Biología Celular y Tisular, Facultad de Medicina, National Autonomous University of Mexico, Mexico City CP 04510, Mexico

**Keywords:** SARS-CoV-2, COVID-19, postmortem samples, lung, inclusion bodies, histological alterations

## Abstract

During the COVID-19 pandemic, SARS-CoV-2 quickly spread all over the world in a pattern of waves. In Mexico, the first wave was from March 2020 to September 2020, and during this time autopsies were forbidden. After that, the postmortem lung samples allowed us to identify histological alterations because of COVID-19. Moreover, SARS-CoV-2 infections are characterized by the manifestation of cytopathic effects like inclusion bodies, and multinucleated cells in alveolar spaces and alveolar walls. Additionally, atypical, enlarged cells, presence of macrophages in alveolar spaces, and congestion of vascular vessels were the other histopathologic alterations of the lung. Our study covered the analysis of nine postmortem lung samples from patients with severe COVID-19 diagnosed by qRT-PCR. The samples were stained with Hematoxylin-Eosin to identify the histological alterations related to lung architecture and cell populations and were subjected to immunohistochemistry for the SARS-CoV-2 Spike and Nucleocapsid proteins. All samples showed alterations associated with diffuse alveolar damage and 1/9 presented no alveolar space, 5/9 presented different levels of pleural fibrosis, and 4/9 presented distention of the small capillaries. Immunohistochemistry results revealed that 4/9 samples showed Spike-positive cytoplasmic inclusion bodies in type I pneumocytes and 2/9 Spike-positive nuclear inclusion bodies in type I pneumocytes. These inclusion bodies were found to be eosinophilic with H&E stains. The H&E results suggest tissue alterations that may contribute to the signs and symptoms of severe COVID-19, as well as the Spike protein expression, as its distribution suggests its participation in pathophysiology.

## 1. Introduction

Coronaviruses (CoV) are respiratory viruses that can cause illnesses ranging from the common cold to severe diseases, such as Middle East Respiratory Syndrome (MERS) and Severe Acute Respiratory Syndrome (SARS) [1]. The recently emerged coronavirus, designated as SARS-CoV-2, was first identified in Wuhan by the Chinese Center for Disease Control and Prevention in December 2019. It is the causal agent of the new coronavirus disease (COVID-19), which spread rapidly throughout China and to six continents in the first quarter of 2020. The WHO declared the COVID-19 pandemic on 11 March 2020 [2,3]. During this time, SARS-CoV-2 quickly spread all over the world in a pattern of waves. In Mexico, the first wave lasted from March 2020 to September 2020 [4,5], during which the Mexican health authorities forbade the performance of autopsies and the study of the different tissue samples obtained from patients who died from severe COVID-19. Severe COVID-19 is characterized by respiratory distress that can either resolve or lead to severe conditions with acute respiratory distress syndrome (ARDS). Recently, some reports described two types of clinical presentation of ARDS. One of them is directly related to acute COVID-19 and is characterized by a classical histopathology pattern of fibrotic diffuse alveolar damage (DAD) or “fibrotic DAD” [6,7], during which the pathologist observed the presence of plasma fibrin in the interstitial and alveolar space, where it polymerizes and produces hyaline membranes [7]. Moreover, DAD is exacerbated by a strong alveolar inflammation caused by inflammatory cells infiltrating the alveoli [6]. During the second phase, it is possible to observe fibroblastic and myofibroblast proliferation and extracellular matrix deposition resulting in fibrosis. The second type of ARDS is related to post-COVID pulmonary fibrosis (PCPF), which includes fibrotic changes, reticular opacities and traction bronchiectasis with or without honeycombing (cyst formation), although there are no strict criteria describing this type of pulmonary fibrosis [6]. Furthermore, extrapulmonary manifestations (cardiovascular complications, gastrointestinal symptoms, renal dysfunction, and neurological manifestations) may occur, leading to the patient’s death [8,9]. The pulmonary damage caused by SARS-CoV-2 is associated with the excessive inflammatory response because of infiltration, activation, and/or depletion of innate and adaptive immune cells [10]. Moreover, severe tissue destruction and thromboembolic events are present in all deadly COVID-19 lung infections [10]. Postmortem lung samples can help us identify alterations that are crucial to understanding the disease’s evolution, virus spread, cell tropism, and the pathophysiological mechanisms of infection [8].

The SARS-CoV-2 is classified within the order *Nidovirales*, belonging to the family *Coronaviridae*, the subfamily *Orthocoronavirinae*, and the genus *Betacoronavirus*. These are enveloped viruses with a protein helical capsid that protects a positive sense, linear RNA molecule. The viral genome codes for four structural proteins (S, M, N, and E), sixteen nonstructural proteins (NS1-16) [11], and nine accessory proteins. The Spike protein (S), in particular, is responsible for the virus’s attachment and subsequent entry into the host cell. The primary site of infection for SARS-CoV-2 is the respiratory epithelial cells, utilizing the angiotensin converting enzyme 2 (ACE2) as its primary receptor. This receptor is found in high concentrations on the epithelial cells of the nose, throat, and, crucially, the lungs’ ciliated cells, Clara, or cylindrical non-ciliated bronchiolar cells, and type II pneumocytes [12]. The SARS-CoV-2 infection produces inclusion bodies, both cytoplasmic and nuclear, as a cytopathic effect; these inclusion bodies are associated with viral replication sites and/or viral protein aggregates. The accumulation of viral proteins in the nucleus might cause the marginalization of chromatin to the nuclear periphery [13]. The identification and knowledge of lung histopathological changes in SARS-CoV-2 infection could be a complementary tool useful for the clinical diagnosis of COVID-19 and contribute to the knowledge of health workers aiming to identify the severe complications of SARS-CoV-2 infection, even when the molecular detection has not yet been performed. Therefore, the purpose of this study is to identify the histopathological changes to detect and localize the viral proteins (Spike and Nucleocapsid) in the postmortem lung samples from Mexican patients with severe COVID-19.

## 2. Results

### 2.1. Histopathologic Findings of Lung Tissue Postmortem Samples from Mexican Patients with Severe COVID-19

A summary of the histopathological findings and other miscellaneous features in postmortem lung samples is demonstrated in Table 1. Histopathological evidence of DAD with decreased vascular perfusion was observed in 100% of samples (9/9), leukocyte infiltration was present in 100% of samples (9/9 samples), among which, samples 2 and 8 (22.2% of samples) resulted in acute inflammatory infiltration (mainly neutrophils) and the rest of the samples (77.7%; samples 1, 3–7, 9) resulted in chronic inflammatory infiltration (mainly mononuclear cells). All the samples (9/9; 100% of samples) showed varying degrees of mononuclear infiltration. The histological analysis showed that 100% of the samples (9/9) presented type I and type II pneumocyte damage, except sample 1, which did not present type II pneumocyte damage; only sample 2 showed metaplasia in type II pneumocytes. Other findings were the hyperplasia and vacuolization of type II pneumocytes in 66.6% of samples (6/9; samples 2, 3, 4, 5, 6, and 8). Sample 3 presented an accumulation of macrophages in the alveolar space. Related to the macrophage population, we noted an increase in the number of these cells with carbon inclusions in 88.8% of samples (8/9; samples 1, 2, 3, 4, 5, 6, 7, and 9); the presence of carbon inclusions might be associated with some events like smoking, air pollution, and in our country, a wide female population still using wood burning stoves to cook. One of the major findings in deadly COVID-19 was fibrosis in 100% of samples. Four samples (44.4% of samples; samples 1, 3, 4, and 7) presented three fibrosis locations: perivascular, pleural, and in the alveolar wall. Samples 5, 6, and 8 (33.3% of samples) showed perivascular and alveolar fibrosis; samples 2 and 9 only showed pleural and alveolar fibrosis, respectively. Detachment of alveolar epithelial cells was identified in 66.6% of samples (6/9; samples 2, 6–9), endothelial and capillary damage were present in 88.8% of samples (only sample 4 did not show these findings), and pulmonary and intravascular dead cells were observed in samples 3 and 5 (2/9, 22.2% of samples). In addition, binucleated and multinucleated cells were observed in 100% of samples (9/9), cytoplasmic and/or nuclear eosinophilic inclusions in 44.4% of samples (4/9), and collapsed alveoli only in 11.1% of samples (1/9). Clots and intravascular protein aggregates (fibrin) in 77.7% of samples (7/9; samples 1, 4, 5, 6, 7, 8, and 9) were also identified.

### 2.2. Histopathological Changes in Deadly COVID-19 Lungs by Hematoxylin and Eosin (H&E) Staining

**Figure 1:**
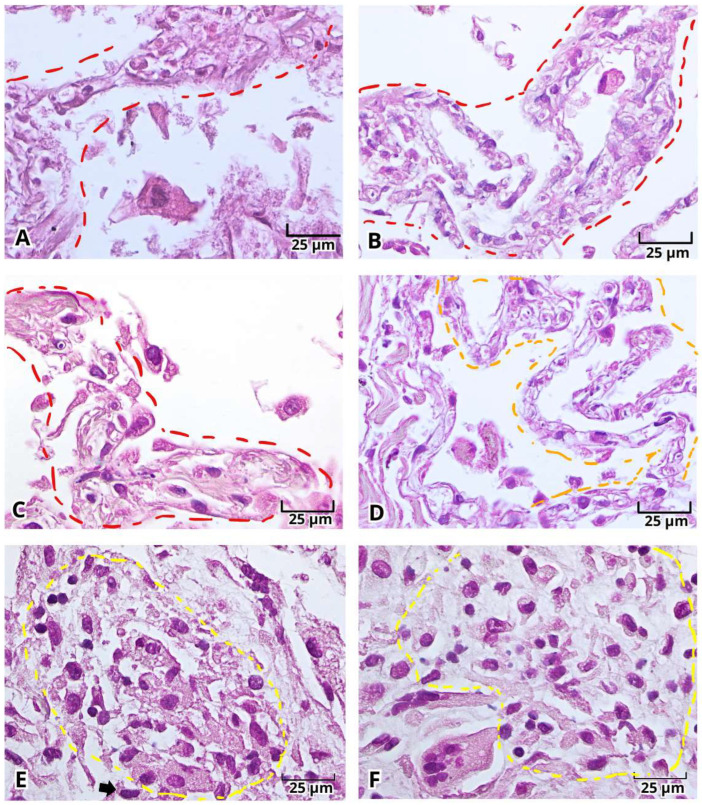
Photomicrographs representative of alveolar fibrosis, capillary dilation, and focal leukocyte infiltration in the histopathological analysis of lung postmortem samples of patients with severe COVID-19. Alveolar wall fibrosis (**A**–**C**, red circle), capillary dilation (**D**, orange circle), leukocyte infiltration (**E**,**F**, yellow circle) and multinucleated cell (**E**, dark arrow). Magnification 400×, the scale bar represents 25 μm.

**Figure 2:**
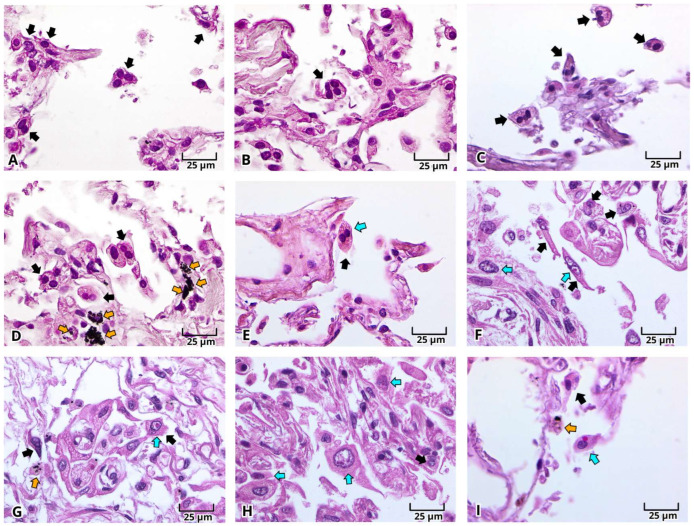
Photomicrographs representative of multinucleated and cytoplasmic/nuclear eosinophilic inclusion bodies cells in histopathological analysis of lung postmortem samples of patients with severe COVID-19. Multinucleated cells (**A**–**I**, dark arrow), carbon inclusions (**D**,**G**,**I**, orange arrow), and cells with cytoplasmic/nuclear inclusion (**E**–**I**, cyan arrow). Magnification 400×, the scale bar represents 25 μm.

**Figure 3:**
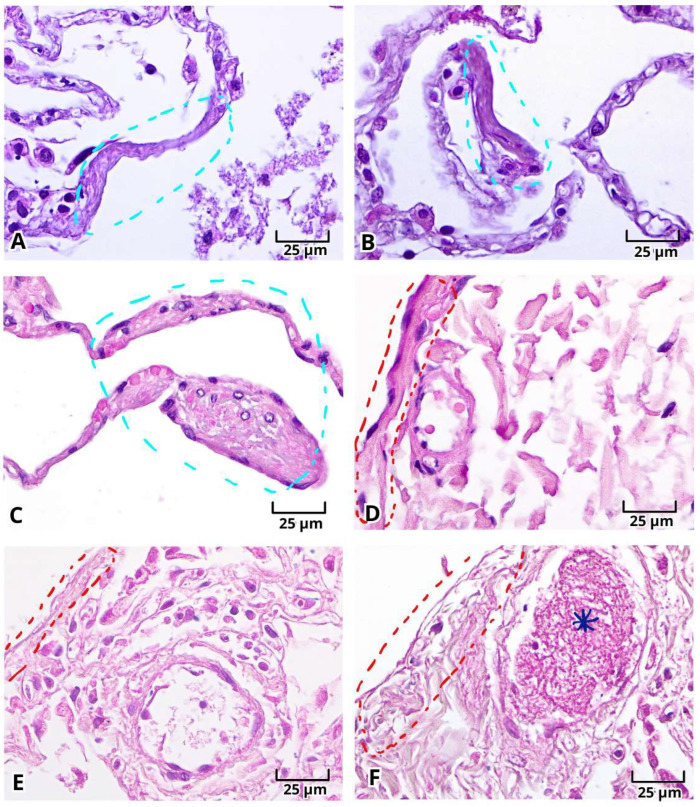
Photomicrographs representative of hyaline membrane and pleural fibrosis in histopathological analysis of lung postmortem samples of patients with severe COVID-19. Hyaline membrane (**A**–**C**, cyan circle), pleural fibrosis (**D**–**F**, red circle), and clot/fibrin protein inside vessels (**F**, blue asterisk). Magnification 400×, the scale bar represents 25 μm.

**Figure 4:**
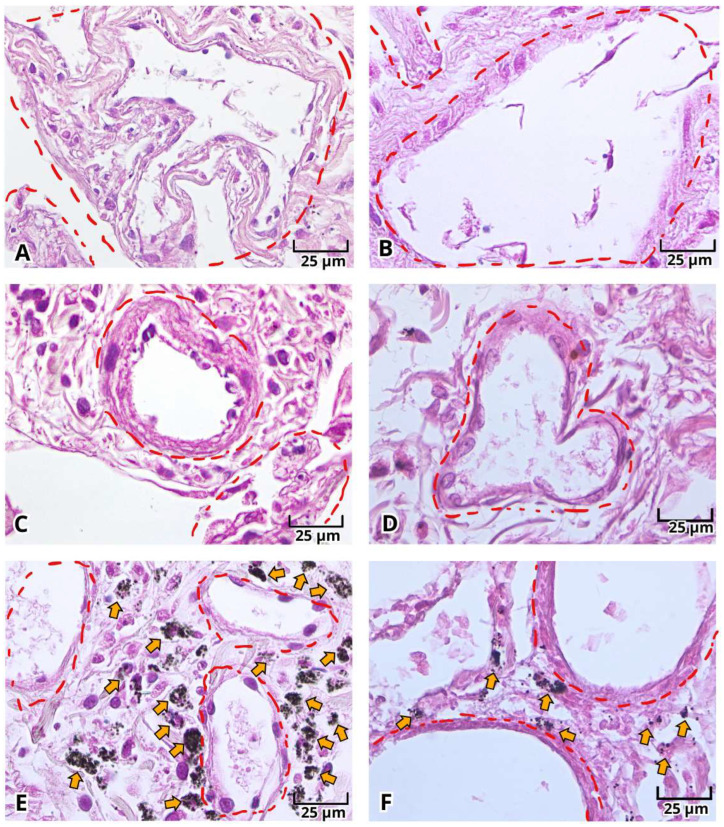
Photomicrographs representative of perivascular damage in histopathological analysis of lung postmortem samples of patients with severe COVID-19. Perivascular damage (**A**–**F**, red circle), carbon inclusions (**E**,**F**, orange arrow). Magnification 400×, the scale bar represents 25 μm.

With H&E staining, we were able to identify the pathological findings in postmortem lung samples. All samples showed alterations associated with DAD (Figure 1) and multinucleated cells (Figure 2A–E and Figure 3A–E). Furthermore, we documented the findings of alveolar (Figure 1A–C, Figure 2A and Figure 3A–E) and vascular fibrosis (Figure 2F–H and Figure 4), as well as different levels of pleural fibrosis (Figure 2F–H). In terms of tissue alterations, we observed distention of the small capillaries (Figure 1D and Figure 3A), alterations in cell populations, and type I and II pneumocytes damage such as: fusion of type II pneumocytes (Figure 1C, Figure 2B–E and Figure 3E), fusion of type I pneumocytes (Figure 1A, Figure 2A,B,D and Figure 3A–D) and eosinophilic cytoplasmic or nuclear inclusions (Figure 3). One of the most frequent findings was the presence of exogenous carbon inclusions, which could be associated with pollution, smoking or the prevalent habit of using wood stoves for cooking (Figure 2E, Figure 3C,E and Figure 4E,F).

### 2.3. Immunohistochemistry of the Viral Proteins of SARS-CoV-2: Spike Protein (S) and Nucleocapsid Protein (N) in Lung Postmortem Samples from Mexican Patients with Severe COVID-19

**Figure 5:**
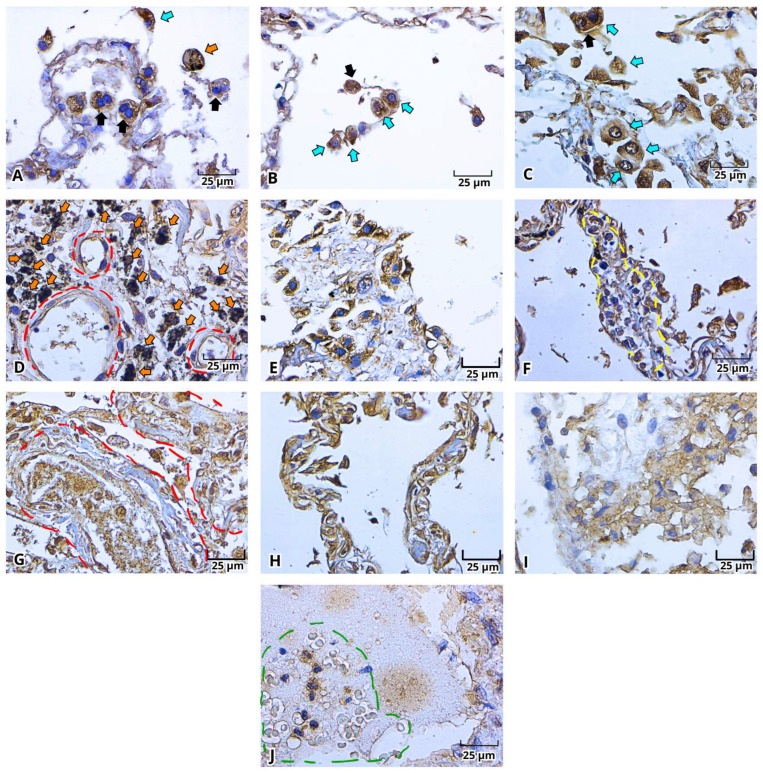
Immunohistochemistry of the Spike (S) protein of SARS-CoV-2 in lung postmortem samples from Mexican patients with severe COVID-19. S protein staining (**A**–**J**, cells and proteins with positive signal in dark brown), cells with cytoplasmatic brown inclusions (**A**–**C**, cyan arrows), multinucleated cells (**A**–**C**, dark arrow), carbon inclusions (**A**,**D**, orange arrow), perivascular (**D**,**G**, red circle), leukocyte infiltration (**F**, yellow circle), and presence of erythrocytes (**J**, dark green circle). Magnification 400×, the scale bar represents 25 μm.

**Figure 6:**
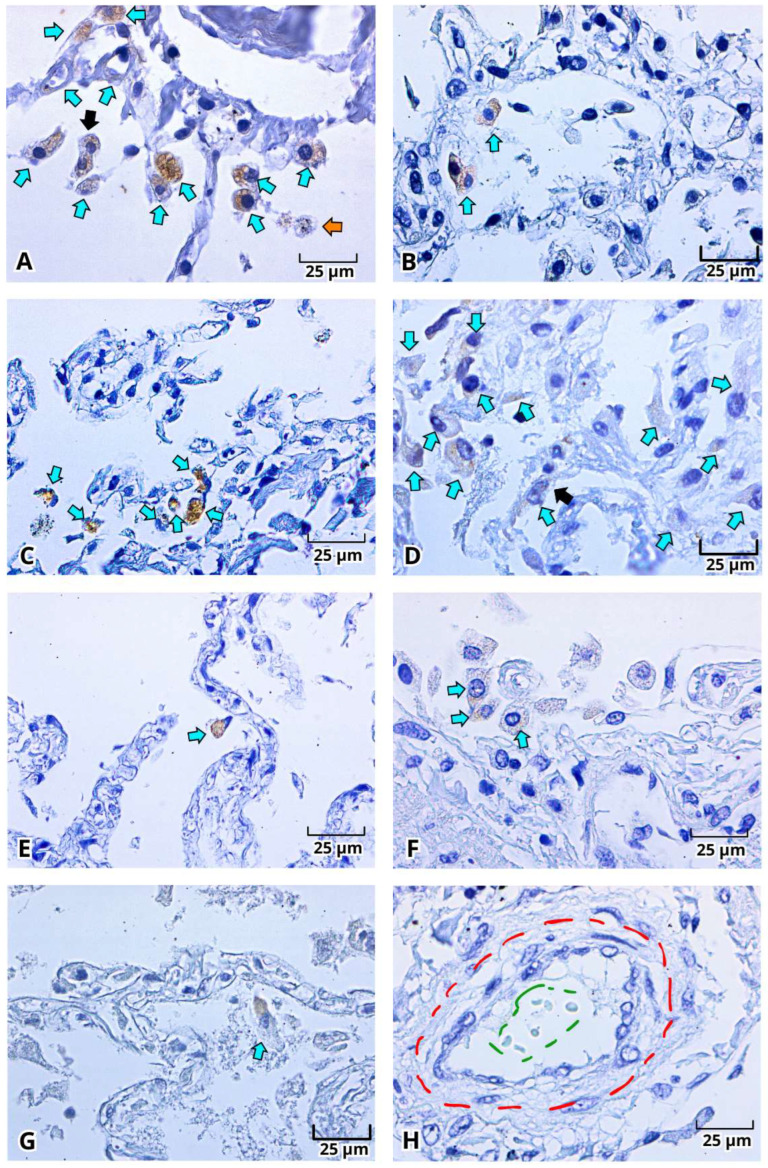
Immunohistochemistry of the Nucleocapsid (N) protein of SARS-CoV-2 in lung postmortem samples from Mexican patients with severe COVID-19. N protein staining: Cells with positive signal (**A**–**G**, cyan arrows), perivascular fibrosis (**H**, red circle), carbon inclusions (**A**, orange arrow), multinucleated cells (**A**,**D**, dark arrow), and presence of erythrocytes (**H**, dark green circle). Magnification 400×, the scale bar represents 25 μm.

**Figure 7:**
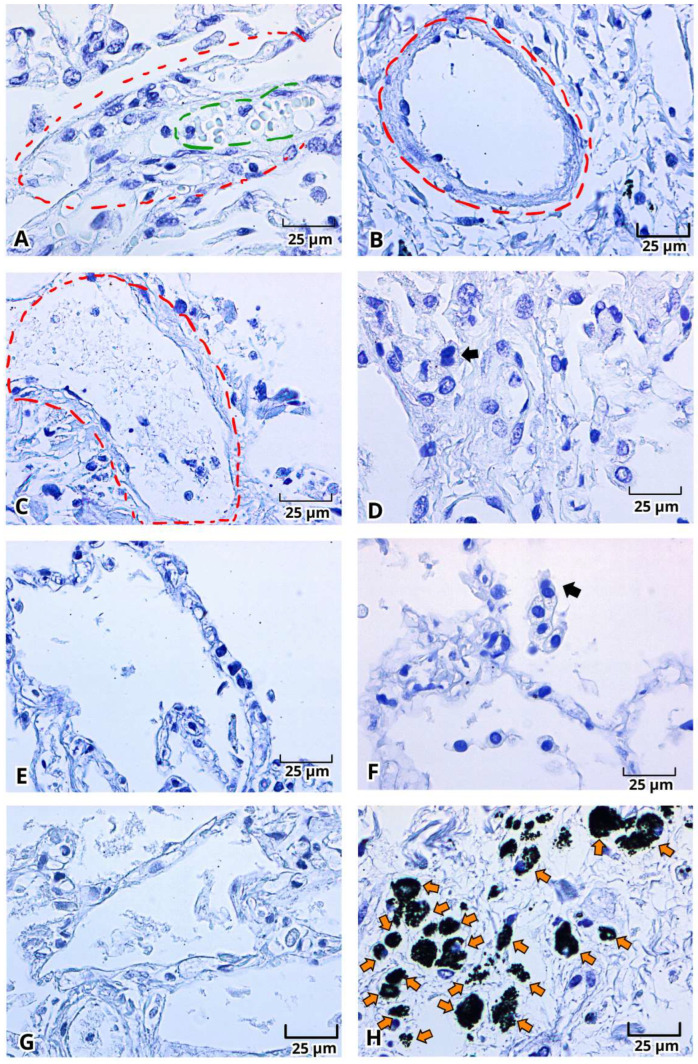
Negative control for immunohistochemistry of the viral proteins of SARS-CoV-2 in lung postmortem samples from Mexican patients with severe COVID-19. Presence of erythrocytes (**A**, dark green circle), perivascular fibrosis (**A**–**C**, red circle), multinucleated cells (**D**,**F**, dark arrow), capillary dilation (**E**,**G**) and carbon inclusions (**H**, orange arrow). Magnification 400×, the scale bar represents 25 μm.

The histopathological findings with H&E staining, indicative of DAD and the presence of multinucleated cells and eosinophilic inclusion bodies, suggested the presence of a viral infection. Accordingly, SARS-CoV-2 viral antigens, particularly the S and N proteins, were identified using enzymatic immunohistochemistry in lung postmortem samples from Mexican patients diagnosed with severe COVID-19 (Figure 5, Figure 6 and Figure 7). Results demonstrated a strong immunoreactivity (dark brown color) of the S protein in 88.8% of the samples (8/9), mainly distributed in detached fusiform flat epithelial cells (type I pneumocytes), detached spherical vacuolated epithelial cells with rounded central nuclei (type II pneumocytes), many with two or three nuclei, macrophages (spherical and larger than pneumocytes), endothelial cells of microvasculature (arterioles, capillaries, and venules), and in intravascular fibrin and intra-alveolar cell detritus. Notably, cytoplasmic inclusion bodies were S-protein-positive (Figure 5). Dust cells or macrophages with carbon inclusions showed diversity in positive immunoreactivity (Figure 5 and Figure 6). N protein immunoreactivity (Figure 6) was subtly positive in 77.7% of samples (7/9), distributed in macrophages, type I and II pneumocytes, and more faintly positive in the endothelium. Cytoplasmic inclusion bodies, dust cells, and the other S-protein-positive structures were N-protein-negative. Hyaline membranes were negative (Figure 7). Erythrocytes also were negative, but numerous polymorphonuclear leukocytes or granulocytes were immunoreactive to the S protein (Figure 7).

## 3. Discussion

Autopsies have been the cornerstone of pathology for centuries, providing crucial insight into disease processes and their multisystemic repercussions. Their importance to uncovering hidden mechanisms makes them an invaluable resource for guiding clinical practice, particularly with emergent diseases like COVID-19.

In the current article, we report on the postmortem pathology of nine patients with confirmed severe SARS-CoV-2 infection. Results showed different histological findings; thus, this wide range of lung injuries might be correlated with the presence of viral proteins and the comorbidities of the dead patients. Our findings are in line with other studies that reported a great variability of microscopic autopsy findings [14,15], as well as the identification of viral particles in the lung [16].

Alveoli are mostly composed of type I pneumocytes, which have classic squamous epithelial morphology, and type II pneumocytes, which are cuboidal, smaller, and possess organelles called lamellar bodies responsible for secreting alveolar surfactant factor which prevents alveolar collapse during expiration [17]. Pulmonary homeostasis is maintained by resident cells, including epithelial cells, endothelial cells, and leukocytes [18]. Resident alveolar macrophages and alveolar and endothelial cells form an important barrier in the lung [19].

Histopathological findings documented in the present study show that in all the postmortem samples (9/9), DAD and decreased vascular perfusion were found. Samples 3, 4, and 5 were distinctive because they presented a higher variability of lung histological changes. Among these changes, we mention those related to leucocyte and monocyte infiltration; these samples also presented morphological alterations of type I and II pneumocytes. They also presented fibrosis, multinucleated cells, and clots. One of the most relevant histological findings in 7 of 9 samples was the presence of carbon inclusions in macrophages; we do not know of any other report that indicates the presence of carbon inclusions in COVID-19 postmortem samples. We associate the presence of carbon grains with different risk factors such as smoking, carbon inhalation in work environments, air pollution, and the use of wood burning stoves for cooking that contribute significantly to the development of COPD, which could increases the risk of inducing a severe COVID-19 disease that could even lead to death [20,21]. Although only some of the samples presented cytoplasmic and/or nuclear eosinophilic inclusion bodies, which could be associated with the virus presence, we analyzed all the samples to detect the viral proteins, and we were able to identify the viral proteins even in those samples that did not show inclusion bodies. We detected viral proteins by immunohistochemistry (both S and N) in the endothelium, macrophages, and type I and II pneumocytes. The presence of viral particles in the endothelium might be a cause of dysregulation of the clotting system, which particularly affects small vessels and leads to pulmonary microthrombi [9], as presented in 7/9 of our samples with clots in vessels. Previous studies that used transmission and scanning electron microscopy (TEM and SEM) demonstrated the presence of SARS-CoV-2 in lung specimens [9,22]. According to these reports, the presence of S and N protein immunoreactively damaged alveolar cells, macrophages, and endothelial cells, even when the signal of N was lower than S. This implies that in the lung postmortem samples analyzed, the virus was actively replicative during the severe clinical phase of COVID-19, and contributed to the tissue damage, represented by DAD and fibrosis. According to some authors, the histopathology and ultrastructural changes in the lung correlated with the presence of viral particles. The present immunohistochemical results are consistent with a previous report in which the tracheal epithelium was also affected [9].

Finally, COVID-19 is a multifactorial disease, in which different factors such as comorbidities, age, virus replication, and proinflammatory immune response participate in the course of the illness from asymptomatic to severe cases, and even to death. Many pathological studies tried to explain the critical histopathological changes observed in the postmortem samples; all of them agreed with the diversity of changes present not only in the lung but also in other organs like heart, kidney, and gut. In conclusion, this descriptive study reports the histopathological findings in a small series of postmortem lung samples from patients with severe COVID-19, including changes compatible with different stages of diffuse alveolar damage and detectable immunoreactivity to SARS-CoV-2 S and N proteins, without indicating disease specificity or causal associations, which are consistent with two other studies previously published in Mexico.

## 4. Materials and Methods

### 4.1. Lung Tissue Samples

Nine deceased patients with confirmed diagnoses of SARS-CoV-2 infection were accrued in our study. The lung autopsy samples were donated by Instituto Nacional de Enfermedades Respiratorias (INER) from the National Health Secretary. All the samples were clinically diagnosed as COVID-19 and confirmed by qRT-PCR. This study was approved by the ethics committee of the Faculty of Medicine, UNAM. The samples were collected at INER and were conducted with the written consent of the patient’s family members in accordance with the regulations issued by INER Health Commission and the Declaration of Helsinki.

### 4.2. Tissue Paraffin Embedding

The standard histological procedure involves the embedding of fixed tissue specimens in paraffin wax to provide the necessary rigid support for thin sectioning, following standard fixation (buffered paraformaldehyde). The cassettes were systematically dehydrated using an ascending series of ethanol concentrations (50% ethanol; 60% ethanol; 70% ethanol; 80% ethanol; 96% ethanol (first bath); 96% ethanol (second bath); absolute ethanol (first bath); absolute ethanol (second bath); absolute xylene (first bath); and absolute xylene (second bath)), followed by treating with xylene to render it transparent and miscible with the infiltration medium. All the previous steps were performed at room temperature with constant agitation 1 h per sample. Subsequently, the tissue underwent infiltration with paraffin wax under the following conditions: 2 h per bath at 56 °C in paraffin (1), and paraffin (2). Prior to starting the embedding process, a TissueTek^®^ II embedding station (Sakura Finetek USA, Inc., Torrance, CA, USA) was pre-heated to its working temperature. The metal embedding molds were also warmed to 56 °C. Molten paraffin was dispensed into a pre-warmed mold placed on the hot plate of the embedding station. Cassettes were removed one by one from the paraffin oven (56 °C) and carefully opened. The tissue was retrieved and oriented in the mold, which was filled with molten paraffin. The cassette base was then placed on top of the mold, and the entire assembly was transferred to a cold plate. After 5–10 min, the resulting paraffin blocks were evaluated to determine their suitability for sectioning. The embedded paraffin histological sections had a thickness between 2 and 4 µm and were then mounted on electrostatically charged slides for subsequent staining protocols.

### 4.3. Hematoxylin-Eosin Staining

The samples were heated at 56 °C for 30 min to remove paraffin. The next step was the rehydration of the electrocharged slides. Afterwards, they were treated with xylene for 10 min, and submerged 20 times in each of the following reagents that stayed arranged in tandem: absolute xylene (1); absolute xylene (2); absolute ethanol (1); absolute ethanol (2); ethanol at 96% (1); ethanol at 96% (2); ethanol at 80%; ethanol at 70%; ethanol at 60% and finally in distilled water.

The slides were put in Mayer’s Hematoxylin for 5 to 10 min; next they were washed with plenty of distilled water. The slides were dipped 5 times in lithium carbonate to intensify the colors, especially the reds and pinks (H&E). After this process the slides were washed with plenty of distilled water and were counterstained with Eosin for 3 to 5 min. Lastly, they were submerged 20 times in each of the following reagents that stayed arranged in tandem: ethanol at 60%; ethanol at 70%; ethanol at 80%; ethanol at 96% (1); ethanol at 96% (2); absolute ethanol (1); absolute ethanol (2); absolute xylene (1); absolute xylene (2). They were kept in xylene until mounting. One by one, the slides were removed from the xylene, 2 drops of mounting medium were added, and coverslips were applied. The mounting medium was dried for 24 h before observing it under BX50 Olympus microscope (TYO, Minato, Japan) with a digital camera digital camera model 1 Lumenera (Teledyne Lumenera, Richmond, BC, Canada) and Infinity Analyze software (v6.3.0).

### 4.4. Immunohistochemistry Protocol

Sample deparaffinization (Note: the tissue could not be allowed to dry): The slides were heated at 56 °C for 45 min to remove the paraffin. Rehydration of electrocharged slides: The slides were placed in xylene for 10 min. They were dipped approximately 20 times in each of the dehydrating reagents using a staining tray with glass support. The reagents were arranged in tandem, as follows: absolute xylene (1); absolute xylene (2); absolute ethanol (1); absolute ethanol (2); ethanol at 96% (1); ethanol at 96% (2); ethanol at 80%; ethanol at 70%; ethanol at 60%; finally, distilled water.

Antigen retrieval: Citrate buffer was prepared with 9.5 mL of citric acid and 45.5 mL of sodium citrate, and the volume was adjusted to 100 mL with distilled water. The slides were placed in a plastic Coplin jar filled with the citrate buffer. The lid was closed loosely, and the jar was placed in the Oster pressure cooker. The cooker lid was closed and set for 24 min on “Soup mode”. After that, the steam was released from the pressure cooker, and it was opened. The jar was removed and left to cool for 30 min at room temperature. The slides were washed three times with the filtered buffer Tris HCl + 0.1% Triton. Excess water was dried. The endogenous peroxidase was blocked with a solution of hydrogen peroxide at 3% in methanol. The slides were placed in a glass Coplin jar filled with a solution of hydrogen peroxide and were incubated for 30 min at room temperature. Afterward, the slides were washed three times with distilled water and then three times with the filtered buffer Tris HCl + 0.1% Triton; the excess water was dried. A hydrophobic marker was used to outline the sample area for it to be tested in order to identify the viral proteins. First, we blocked the nonspecific bindings with 300 µL of 1% bovine serum albumin in Tris HCl that were added to each slide (or enough to cover the sample area). The slides were incubated for 1 h at 37 °C. After this period, the slides were drained and excess moisture was wiped away, ensuring the tissue did not dry out, except for the controls. The primary antibodies were used at a dilution of 1:100 for the SARS-CoV/SARS-CoV-2 Spike antibody (GeneTex (Irvine, CA, USA), catalog number: GTX632604, clone 1A9, made in mouse, Lot: 44174) and 1:100 for the SARS-CoV-2 Nucleocapsid antibody (GeneTex) catalog number: GTX135357, polyclonal, made in rabbit, Lot: 43957). The primary antibodies were diluted with Tris HCl with bovine serum albumin at 0.1%, then 300 µL were added to cover the sample area and the slides were incubated overnight at 4 °C. The slides were washed 3 times with Tris HCl, and excess moisture was dried. The hydrophobic marker was used again to outline the sample area. The secondary antibodies were used at a dilution of 1:50 for HRP-Goat Anti-Mouse (IgG + IgA + IgM; H + L) (Zymed (South San Francisco, CA, USA) catalog number: 65-6420, Lot: 495441A) and 1:50 for ZyMax HRP-Goat Anti-Rabbit IgG (H + L) (Zymed, catalog number: 81-6120, Lot: 30979514) were prepared with Tris HCl with bovine serum albumin at 0.1%. Then, 300 µL was added and the slides were incubated at 37 °C for 1 h. Next, the slides were washed 3 times with Tris HCl buffer, and excess moisture was dried. Since the antigen–antibody complex was revealed using 3,3′-Diaminobenzidine (DAB) as the chromogen, it was prepared as follows: Solution A: 2.5 mg of DAB + 5 mL Tris HCl buffer. Solution B: 3 drops of hydrogen peroxide + 7 drops of distilled water. Three drops from solution B were added to solution A. The sample area was covered with the solution and incubated for 1 min, and the reaction was stopped with distilled water. The slides were kept in distilled water. After that, the slides were dipped 20 times in Mayer’s Hematoxylin and then washed with distilled water. Finally, the dehydration process consisted in dipping the slides 20 times in each of the following reagents in the following order: ethanol at 60%; ethanol at 70%; ethanol at 80% ethanol at 96% (1); ethanol at 96% (2): absolute ethanol (1); absolute ethanol (2); absolute xylene (1); absolute xylene (2). The slides were kept in xylene until mounting. Next, the slides were removed from xylene one by one, two drops of mounting medium were added, and coverslips were applied. The mounting medium was left to dry for 24 h before observing it under BX50 Olympus microscope (TYO, Minato, Japan) with a digital camera model 1 Lumenera (Teledyne Lumenera, Richmond, BC, Canada) and Infinity Analyze software (v6.3.0).

## Figures and Tables

**Table 1:** Lung histopathological changes associated with severe COVID-19: Postmortem samples.

Pathological Findings	Samples								
	1	2	3	4	5	6	7	8	9
Diffuse alveolar damage	✓ *	✓ *	✓ *	✓ *	✓ *	✓ *	✓ *	✓ *	✓ *
Decreased vascular perfusion	✓ **	✓	✓	✓	✓	✓	✓	✓	✓ **
Leukocyte infiltration	✓	✓	✓	✓	✓	✓	✓	✓	✓
Type I Pneumocytes damage	✓	✓	✓	✓	✓	✓	✓	✓	✓
Type II Pneumocytes damage	Ø	✓	✓	✓	✓	✓	Ø	Ø	Ø
Type II Pneumocytes metaplasia	Ø	✓	Ø	Ø	Ø	Ø	Ø	Ø	Ø
Increased type II Pneumocytes	Ø	✓	✓	✓	✓	✓	✓	✓	Ø
Vacuoles in type II Pneumocytes	Ø	✓	✓	✓	✓	Ø	✓	✓	Ø
Macrophages with Carbon Inclusions	✓	✓	✓	✓	✓	Ø	✓	Ø	✓
Increase in alveolar Macrophages	Ø	Ø	✓	Ø	Ø	Ø	Ø	Ø	Ø
Perivascular fibrosis	✓	Ø	✓	✓	✓	✓	✓	✓	Ø
Pleural fibrosis	✓	✓	✓ ^a^	✓ ^b^	Ø	Ø	✓	Ø	Ø
Alveolar wall fibrosis	✓	✓	✓	✓	✓	✓	✓	✓	✓
Capillary dilation	✓	✓	✓	✓	✓	Ø	✓	Ø	Ø
Detachment of vascular endothelium	Ø	Ø	✓	✓	✓	Ø	Ø	Ø	Ø
Epithelial Cell Detachment	✓	✓	✓	✓	✓	✓	Ø	✓	✓
Alveolar and Vascular cell detritus	✓	✓	✓	✓	✓	✓	Ø	✓	✓
Intravascular dead cells	Ø	Ø	✓	✓	✓	✓	✓	✓	✓
Multinucleated cells	✓	✓	Ø	✓	✓	✓	✓	✓	✓
Clot/fibrin inside the blood vessels	✓	Ø	Ø	✓	✓	✓	✓	✓	✓
Collapsed alveoli	Ø	Ø	Ø	Ø	Ø	Ø	Ø	✓	Ø
Cytoplasmic/Nuclear eosinophilic inclusion bodies	✓	Ø	✓	Ø	✓	Ø	✓	Ø	Ø

✓ Presence. * Proliferative and fibrotic phase. ** Areas with different degrees of perfusion. ^a^ Without pleural fibrosis. ^b^ Fibrin protein content. Ø Scarce or absent.

## Data Availability

The original contributions presented in this study are included in the article. Further inquiries can be directed to the corresponding authors.

## Abbreviations

The following abbreviations are used in this manuscript:

COVID-19	Coronavirus Disease 2019
SARS	Severe Acute Respiratory Syndrome
MERS	Middle East Respiratory Syndrome
CoV	Coronaviruses
SARS-CoV-2	Severe Acute Respiratory Syndrome Coronavirus 2
H&E	Hematoxylin and Eosin staining
qRT-PCR	Real-Time Polymerase Chain Reaction.
ARDS	Acute Respiratory Distress Syndrome
DAD	Diffuse Alveolar Damage or Fibrotic DAD
PCPF	Post-COVID Pulmonary Fibrosis
S	Spike glycoprotein
N	Nucleocapsid protein
ACE2	Angiotensin Converting Enzyme 2
COPD	Chronic Obstructive Pulmonary Disease
TEM	Transmission Electron Microscopy
SEM	Scanning Electron Microscopy
INER	Instituto Nacional de Enfermedades Respiratorias
DAB	3,3′-Diaminobenzidine
